# Efficacy of a Prescription-Based Mobile Digital Therapeutic as an Adjunct to Pharmacotherapy for the Acute Phase of Panic Disorder: Multicenter Randomized Controlled Trial

**DOI:** 10.2196/101125

**Published:** 2026-10-05

**Authors:** Yujin Ko, Junhyung Kim, Sunyoung Park, Hyunkyu Kim, June-ho Seo, Il Ho Park, Jeemin Lee, Jae-Jin Kim

**Affiliations:** 1 Department of Psychiatry, Soonchunhyang University Bucheon Hospital, Bucheon, Republic of Korea; 2 Department of Psychiatry, Kangbuk Samsung Hospital, Sungkyunkwan University School of Medicine, Seoul, Republic of Korea; 3 Department of Psychiatry, National Health Insurance Ilsan Hospital, Goyang, Republic of Korea; 4 Department of Psychiatry, Yonsei University Wonju Severance Christian Hospital, Wonju, Republic of Korea; 5 Department of Psychiatry, Catholic Kwandong University, International St. Mary's Hospital, Incheon, Republic of Korea; 6 Institute of Behavioral Sciences in Medicine, Yonsei University College of Medicine, Seoul, Republic of Korea; 7 Department of Psychiatry, Yonsei University Gangnam Severance Hospital, 211 Eonju-ro, Gangnam-gu, Seoul, 06273, Republic of Korea, 82 2-2019-3341

**Keywords:** acute-phase panic disorder, digital therapeutic, self-guided therapy, randomized controlled trial, mobile health

## Abstract

**Background:**

Although pharmacotherapy is the primary treatment for patients with acute-phase panic disorder, its incomplete efficacy causes them to experience frequent panic attacks and severe anticipatory anxiety for a considerable period. A prescription-based mobile digital therapeutic (DTx) that integrates self-guided cognitive behavioral therapy (CBT), real-time symptom management, and lifestyle tracking can be used as an adjunct to pharmacotherapy for these patients, helping them achieve rapid symptom recovery.

**Objective:**

This study aimed to evaluate the efficacy of this adjunctive DTx in alleviating symptoms in patients with acute-phase panic disorder.

**Methods:**

In total, 66 acute-phase patients experiencing frequent panic attacks were recruited from 6 institutions and randomly divided into a group receiving pharmacotherapy combined with DTx or pharmacotherapy alone, participating in an 8-week multicenter single-blind trial. The DTx app included 3 major services: training service consisting of structured CBT modules, companion service of just-in-time modules for coping with panic attacks, and care service providing daily self-management tracking tools. The self-report scales used as efficacy indicators were administered via paper questionnaires during a total of 4 visits at baseline, week 2, week 4, and week 8. The primary end point, the change in the Panic Disorder Severity Scale-Self Report (PDSS-SR) score, and the secondary end points, such as changes in overall anxiety, depression, panic-related catastrophic cognitions, and fear of bodily sensations, were compared between the 2 groups. Adherence to the DTx was objectively assessed using device use metrics.

**Results:**

As 5 enrolled patients were excluded due to insufficient safety analysis or efficacy evaluation, the final analysis included 32 in the DTx group and 29 in the control group. The DTx group showed a significantly greater reduction in PDSS-SR scores at week 8 compared to the control group (mean 40.29%, SD 26.68% vs mean 17.61%, SD 30.37%; *P*=.007). This therapeutic benefit appeared rapidly by week 2 (mean 26.66%, SD 22.03% vs mean 11.05%, SD 21.73%; *P*=.02). The responder (≥40% PDSS-SR reduction) rate was also significantly higher in the DTx group (17/32, 53.12% vs 5/29, 17.24%; *P*=.007). While changes in depression and somatic fears were transient or similar between groups, the DTx group showed significantly greater sustained improvements in overall anxiety and catastrophic cognitions. The overall mean adherence rate was 78.12%, with no significant difference between responders and nonresponders.

**Conclusions:**

The adjunctive use of our prescription-based DTx resulted in early and sustained symptom reductions across panic severity, overall anxiety, and catastrophic cognitions, suggesting that this multifunctional and self-guided DTx including just-in-time management and lifestyle tracking can serve as a practical complement to routine psychiatric care for acute-phase patients experiencing frequent panic attacks. This app is expected to present a new framework for the treatment of acute-phase panic disorder by enabling the incorporation of various symptomatic aspects in patients’ daily life into clinicians’ evaluations and guidance in the clinic.

## Introduction

Panic disorder is characterized by recurrent, unexpected panic attacks accompanied by persistent concern about further attacks and maladaptive behavioral changes [1]. These attacks manifest as abrupt surges of intense fear, typically involving severe physical and cognitive symptoms such as palpitations, shortness of breath, dizziness, and a profound fear of dying or losing control. These acute episodes are highly distressing, often leading patients to seek emergency medical care due to the catastrophic misinterpretation of normal somatic sensations as signs of imminent physical or mental harm [2]. Consequently, the frequent recurrence of these attacks triggers severe anticipatory anxiety and agoraphobic avoidance, causing patients to desperately try to prevent future episodes. Together, these symptoms can lead to substantial functional impairment, restricting mobility, work productivity, and social participation [3]. Epidemiologic studies indicate that panic disorder is relatively common in the general population, with approximately 2%‐3% for 12-month prevalence and 4%‐5% for lifetime prevalence in adults [4]. Given its substantial clinical burden and functional impairment, considerable research has focused on improving treatment strategies for panic disorder, and effective, scalable interventions that can alleviate frequent acute attacks remain an ongoing clinical need [5].

The most common method of treating panic disorder is pharmacotherapy, and selective serotonin reuptake inhibitors (SSRIs) and benzodiazepines are widely recommended as a first-line pharmacological option [6]. SSRIs provide effective long-term reduction in panic symptoms and relapse risk with relatively favorable safety, but their clinical benefits are delayed, whereas benzodiazepines produce rapid relief of panic symptoms, but their use is limited by sedation, cognitive impairment, and significant risks of dependence [7]. Despite pharmacotherapy, the remission rate of panic disorder in randomized controlled trials is generally around 30%‐50% [8], meaning that a significant number of patients do not achieve complete remission. Moreover, even for patients who have achieved remission, it takes a considerable amount of time to reach that state. Consequently, many patients in the acute phase of panic disorder continue to experience unpredictable and distressing panic attacks despite receiving active medication. Therefore, there is a need for optimized, accessible adjunctive interventions that can provide immediate support to better manage the recurrence of these acute episodes.

Cognitive behavioral therapy (CBT), provided by a skilled therapist, may be considered one of these interventions and is particularly well-suited to panic disorder, in that it directly targets the maintenance mechanisms that trigger and exacerbate frequent acute panic attacks, namely, catastrophic misinterpretation of bodily sensations, anticipatory anxiety, and avoidance behaviors [9]. The core components of CBT, including psychoeducation, cognitive restructuring, and exposure exercises, systematically reduce safety behaviors and increase adaptive coping, producing durable improvements in both the frequency of acute attacks and functional impairment [10], and the addition of digital content to these therapeutic techniques provides additional benefits to the treatment of panic disorder [11,12]. However, despite robust evidence supporting the effectiveness of CBT for panic disorder, access to adequate CBT in routine practice remains limited. Delivering CBT with sufficient intensity and fidelity typically requires trained therapists, multiple sessions, and sustained engagement, but these resources are often constrained by availability, cost, and scheduling barriers [13]. Consequently, many patients receive only pharmacotherapy and brief psychoeducation without sufficient opportunities for structured skills training, guided exposure, or ongoing reinforcement outside the clinic [14].

In the treatment of panic disorder, patients’ everyday self-management and lifestyle factors are important considerations. Patients often report that panic symptoms are worsened by common, modifiable triggers such as caffeine intake, irregular sleep, alcohol or nicotine use, and physical deconditioning [15-17]. While behavioral changes can support anxiety regulation, it is difficult for patients struggling with unpredictable panic episodes to implement them on their own in daily life. In routine outpatient care, lifestyle counseling is frequently delivered as brief, one-off advice (eg, “avoid caffeine” or “exercise regularly”) during time-limited visits, and clinicians may not be able to address these topics consistently at every visit. Moreover, many relevant behaviors are highly variable between visits and difficult to track without structured tools, making it challenging to provide timely, individualized feedback. As a result, although lifestyle-focused self-management may represent an important, patient-empowering component of care (particularly because it emphasizes skills and behaviors that patients can actively practice) [18], real-world implementation remains constrained in standard clinic-based settings.

To bridge the gap between evidence-based nonpharmacological care and real-world delivery, a range of self-help and technology-assisted strategies have emerged, including bibliotherapy, web-based programs, and app-based interventions [19,20]. In this context, digital therapeutics (DTx) have gained increasing attention as a scalable approach to deliver structured, protocol-driven interventions beyond the constraints of face-to-face therapy. In particular, regarding the effectiveness of CBT, which is a core component of most DTx, self-guided methods using digital technology have been reported to be noninferior to face-to-face methods provided by a therapist in terms of improving various medical conditions [21-23]. By offering standardized content, on-demand access, and the ability to provide repetitive practice and monitoring even outside the clinic, DTx holds promise for expanding the reach of nonpharmacological treatment for panic disorder [24].

However, translating this promise into consistent clinical benefits often requires a multifaceted approach that takes into account the complex, fluctuating nature of panic disorder. Currently available digital interventions tend to excel in specific therapeutic domains. For instance, Freespira, a well-known prescription-based DTx, provides a capnometry-guided respiratory intervention to regulate physiological panic symptoms [25]. Similarly, established transdiagnostic digital CBT programs targeting anxiety disorders, such as Velibra and FearFighter, offer structured cognitive restructuring and exposure modules [26,27], and digital modules specific to panic disorder have also been introduced in the form of exposure therapy and self-guided mindfulness training [28]. While these targeted approaches demonstrate clinical value, managing the full spectrum of panic disorder often invites a more integrated strategy. Existing modular programs may not fully capture the need for real-time, context-responsive support, such as ecological momentary interventions and just-in-time adaptive interventions (JITAIs), which are critical when patients face abrupt anticipatory anxiety or early panic [29-31]. In addition, they have not fully used ongoing opportunities, such as incorporating practical tools for daily lifestyle tracking within self-directed applications [32] or integrating with routine clinical care, including progress reviews and skill reinforcement through the participation of health care professionals [33]. These considerations suggest that DTx for panic disorder may benefit from an integrated framework that combines structured CBT training, ongoing self-management support, context-responsive tools for symptom episodes, and linkage to standard clinical settings.

In line with these considerations, we developed a prescription-based mobile DTx for panic disorder that integrates multiple components within a single protocol [34]. This DTx app was designed based on CBT principles and relevant guidelines [35] and is delivered via a smartphone and a smartwatch. Specifically, this comprises 3 patient-facing services and a clinician-facing management console. The training service provides structured, CBT-oriented modules that include psychoeducation, cognitive restructuring to address catastrophic misinterpretations of bodily sensations, and guided interoceptive and in vivo exposure exercises. The companion service delivers JITAIs offering immediate tools such as paced breathing and grounding techniques during periods of acute symptom escalation or anticipatory anxiety. The care service facilitates daily self-management and lifestyle modifications by allowing patients to monitor mood, sleep patterns, exercise levels, and potential triggers such as caffeine and alcohol intake and by providing individualized feedback based on accumulated data. The management console allows clinicians to monitor patients’ use and adherence to the 3 services in real time, facilitating linkage between digital engagement and routine clinical care.

The purpose of this study was to verify, through a randomized controlled clinical trial, whether this DTx provides additional clinical benefits when added to conventional pharmacotherapy compared with conventional pharmacotherapy alone during an 8-week treatment period in acute-phase patients experiencing frequent panic attacks. The primary outcome of the efficacy evaluation was to assess changes in overall panic symptom severity. Because panic disorder is often accompanied by broader emotional symptoms and fear-related cognitive and somatic processes, secondary outcomes were to assess changes in overall anxiety and depression, panic-related catastrophic cognitions, and the fear of bodily sensations. In addition to the mean symptom change, we aimed to provide a more interpretable measure of treatment benefit by calculating the proportion of patients who achieved a clinically meaningful level of improvement.

## Methods

### Trial Design and Setting

This study was a multicenter, prospective, randomized, and single-blind (clinician-blinded) clinical trial evaluating the efficacy of using prescription-based DTx for 8 weeks as an adjunct to conventional pharmacotherapy in participants with acute-phase panic disorder. The target number of participants was 66, and recruitment was conducted competitively at the psychiatric outpatient clinics of 6 institutions in the Republic of Korea from May 2025 to September 2025. These institutions were all university-affiliated or general hospitals where psychiatrists and clinical psychologists served as primary and assistant therapists, respectively. The target sample size was determined based on prior internet-based CBT trials for panic disorder [36]. Using an expected between-group effect size of Cohen *d*=1.09, with a 2-sided significance level of .05 and 90% power, a minimum of 18 participants per group was required. Accounting for an anticipated dropout rate of up to 45%, the target enrollment was set at 66 participants.

### Ethical Considerations

The trial protocol of this study was approved by the Korea Ministry of Food and Drug Safety (protocol 1825) and the institutional review board of each participating institution (final approval date: April 16, 2025). The trial was registered retrospectively at the Clinical Research Information Service, linked to the WHO International Clinical Trials Registry Platform (KCT0010500; first submission date: April 22, 2025; registration date: May 22, 2025). Of the total 66 participants, 1 was enrolled on May 9, 2025, and the remaining 65 were all enrolled after May 22, 2025. This trial was conducted in compliance with the ethical standards of the Declaration of Helsinki and in full adherence to preregistered methodologies to ensure consistency and fidelity to the original study design (refer to Checklist 1 for the CONSORT-eHEALTH [Consolidated Standards of Reporting Trials of Electronic and Mobile Health Applications and Online Telehealth] checklist). In addition, to strengthen the transparency and clarity of reporting on study results, we adhered to “CONSORT 2025 expanded checklist of information to include when reporting a randomised trial” [37] (refer to Checklist 2). All participants submitted written informed consent for participation in the clinical trial after hearing an explanation of its scope and procedures. The consent form clearly stated the right of participants to withdraw from the trial at any time without penalty and specified that the data would be used for both primary and secondary analyses, would be anonymized prior to analysis for privacy and confidentiality, and that personally identifiable information would be excluded from the analysis dataset. All participants could receive a total of 150,000 KRW (US $104.88 as of April 22, 2025) in financial compensation for participation, by receiving 50,000 KRW (US $34.96 as of April 22, 2025) each at the second, third, and fourth visits.

### Eligibility Criteria

During the recruitment period, eligibility was assessed on patients with panic disorder who visited the clinics participating in the recruitment process, and the general public was not included. The primary inclusion criteria were patients aged 19 years or older diagnosed with panic disorder according to the *Diagnostic and Statistical Manual of Mental Disorders, Fifth Edition* (*DSM-5*) or the *International Classification of Diseases and Related Health Problems, Tenth Edition* (*ICD-10*) who had a total score of ≥11 (“slightly ill” level or worse) and item 1 score ≥2 (“1 or 2 full panic attacks and/or multiple limited symptom attacks per day” level or higher) on the Panic Disorder Severity Scale-Self Report (PDSS-SR) [38]. Additional inclusion criteria were the ability to read and understand Korean and complete questionnaires and the ability to use a smartphone. Exclusion criteria included (1) having a major psychiatric disorder such as schizophrenia spectrum disorder, bipolar disorder, major depressive disorder, or substance use disorder, currently present or in the past; (2) an uncontrolled or severe illness requiring hospitalization or equivalent level of care; (3) having attempted suicide within the past 3 months or being judged by a psychiatrist to be at high risk of suicide; (4) having started or currently receiving CBT within the past 3 months for any reason; and (5) having visual or hearing impairment or cognitive impairment (eg, dementia).

### Randomization and Blinding

Participants were recruited sequentially after applying these inclusion and exclusion criteria, and the recruitment process continued until the target number of participants of 66 was finally reached. They were randomized in a 1:1 ratio using a stratified block randomization scheme at each institution and divided into a group receiving conventional pharmacotherapy with DTx added (referred to as the DTx group) and a group receiving conventional pharmacotherapy alone (referred to as the control group). Randomization was performed using a computer-generated randomization schedule via an Interactive Web Response System integrated within the Electronic Data Capture system.

While participants were aware of their assigned group due to the nature of the digital intervention, the clinicians determining the type and dosage of medication were blinded to the group allocations. Conventional pharmacotherapy was delivered in a naturalistic clinical setting, where clinicians could adjust psychiatric medications (eg, SSRIs or benzodiazepines) for each participant as clinically necessary, just as in real-world outpatient care. Both groups made a total of 4 visits over an 8-week period (weeks 0, 2, 4, and 8). During each visit, they received outpatient care from their attending clinician and participated in the symptom severity assessment in a separate laboratory.

### Intervention

The DTx app for patients with panic disorder (product name: Paniclean, manufacturer: Waycen Inc) can be installed on their smartphones using a personal login code provided by prescription and runs on both iOS and Android. This app does not contain information about institutions, such as hospitals or universities, that participated in its development and consists of 3 major services designed for patients to run themselves at any time for symptom management in their daily lives, such as training, companion, and care, and a service assistance section (Figure 1A). Training service includes structured CBT modules, such as “Studying about Panic,” “Correcting Cognitive Distortions,” “Diaphragmatic Breathing,” “Progressive Muscle Relaxation,’ “Interoceptive Exposure,” “Mindfulness,” and “Real-life Exposure.” Companion service comprises 2 just-in-time modules of “Preventing a Panic Attack” and “During a Panic Attack,” featuring listening to calming words, breathing or relaxation guide, panic prevention or escape guide, and a talk to chatbot function. Modules of care service as daily self-management tracking tools consist of “Medicine Record,” “Sleep Record,” “Exercise Record,” and “Life-style Habits Record.” Modules of service assistance are “Reports and Feedback,” “More Information,” and “Frequently Asked Questions.” Participants received face-to-face instructions on how to use the app themselves from an assistant therapist, rather than the clinician who prescribed the medication. As shown in Figure 1B, companion and care services were available at any time during the trial period, but training service was prescribed by the assistant therapist at each visit to allow for the step-by-step use of only the designated modules on a weekly basis, with access to all content only available by the final visit. Participants were instructed to complete all training service modules assigned to the relevant week, use companion service at least once a week, and record care service modules daily if possible. Aside from the automatic notifications built into the app, no additional prompts or reminders related to DTx use were used during the trial period.

**Figure 1.**
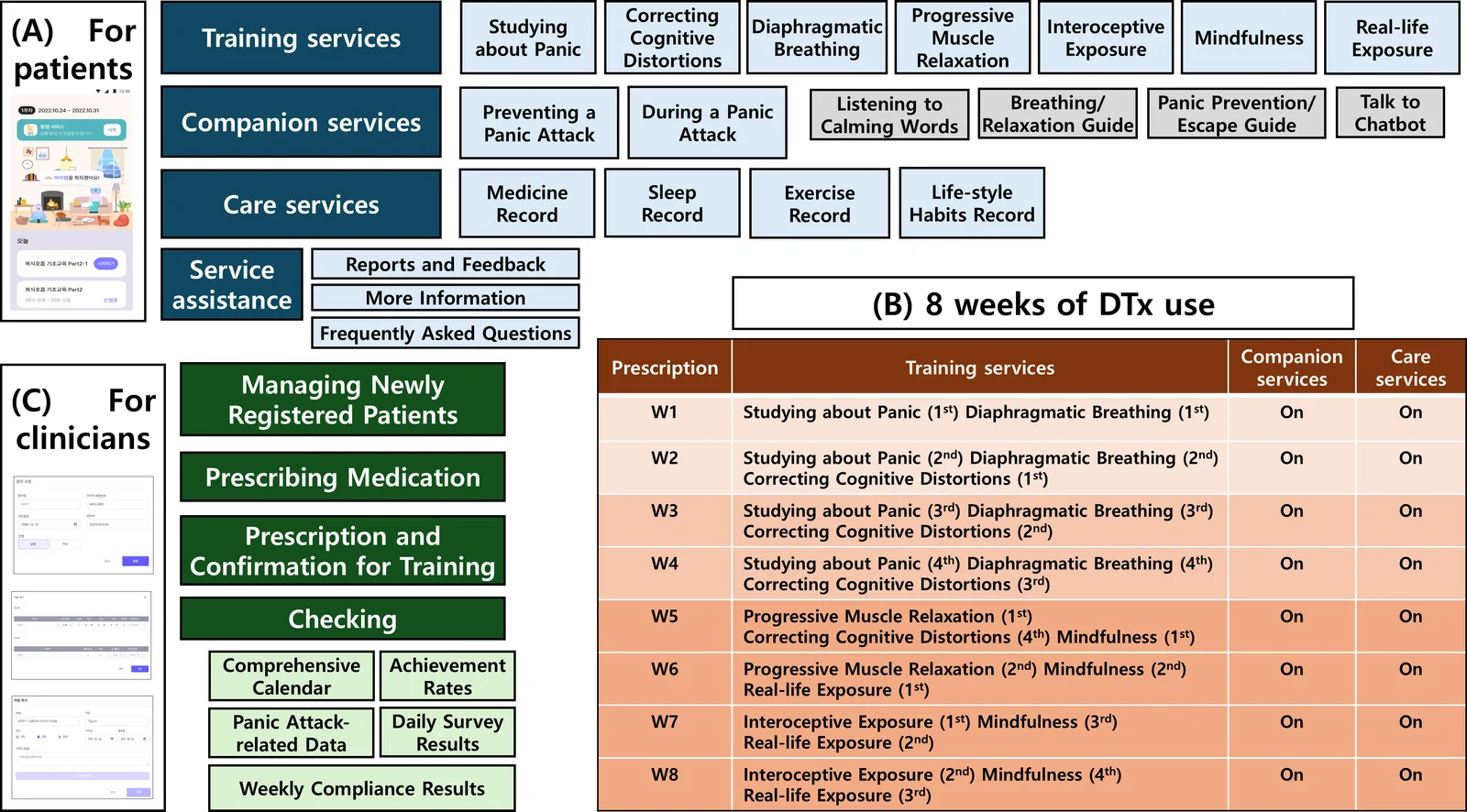
Overview of the prescription-based DTx app. (A) Patient-facing app structure including training, companion service, care service, and service assistance. These consist of 7 modules, 2 modules with 4 submodules, 4 modules, and 3 modules, respectively. (B) Weekly schedule of the services prescribed during the 8-week intervention period. Training services provide cognitive behavioral therapy modules sequentially on a weekly basis, whereas companionship and care services are available for use at any time throughout the 8 weeks. (C) Structure of the management console for the clinicians, consisting of 4 modules, 1 of which has 5 submodules. DTx: digital therapeutic.

The management console is a web-based tool through which clinicians can prescribe use of the app, check patient records, and provide feedback. This consists of various modules, such as “Managing Newly Registered Patients,” “Prescribing Medication,” “Prescription and Confirmation for Training,” “Checking: Comprehensive Calendar,” “Checking: Achievement Rates,” “Checking: Panic Attack-related Data,” “Checking: Daily Survey Results,” and “Checking: Weekly Compliance Results” (Figure 1C). During the clinical trial, this management console was used by the assistant therapist at every visit to monitor the participant’s app use status and provide necessary advice, and then prescribe modules of training service to be performed by the next visit. Further details regarding interface elements, user workflows, and screenshots for the app and management console have been fully described in our previous usability study [34], and their content was frozen without upgrades during the clinical trial.

### Outcomes

The psychological scales used to evaluate the primary and secondary outcome variables as efficacy indicators were all self-report and were administered via paper questionnaires during a total of 4 visits over baseline, week 2, week 4, and week 8. The primary outcome variable was the change in PDSS-SR scores according to the duration of DTx use. The PDSS-SR is a 7-item self-report measure of panic disorder severity capturing key domains such as panic frequency and distress, anticipatory anxiety, avoidance, and functional impairment; each item is rated on a 5-point Likert scale ranging from 0 to 4 with varying descriptors [38], yielding a total score between 0 and 28. The total scores of this scale were categorized as mild (≤10), moderate-to-severe (11-15), and severe (16-28) [39]. Furthermore, to evaluate the relative degree of symptom improvement, the percentage reduction in PDSS-SR scores from baseline to each follow-up visit (weeks 2, 4, and 8) was also calculated and compared between the groups. To complement the mean change with a clinically interpretable metric, we also evaluated a responder outcome. A responder was defined as achieving a ≥40% reduction in the PDSS-SR total score from baseline to week 8, a validated threshold representing a clinically meaningful therapeutic response [40].

Secondary outcome variables were the changes in scores on 3 additional scales measuring overall anxiety and depression, panic-related cognitions, and fear of bodily sensations from baseline to each follow-up visit (weeks 2, 4, and 8). Overall anxiety and depression were assessed using the Hospital Anxiety and Depression Scale (HADS). The HADS comprises 14 items divided into two 7-item subscales (anxiety and depression). Each item is rated on a 4-point Likert scale ranging from 0 to 3 with varying descriptors, resulting in a total score of 0 to 21 for each subscale, with higher scores indicating greater symptom severity [41]. Panic-related catastrophic cognitions were evaluated using the Agoraphobic Cognitions Questionnaire (ACQ), which consists of 14 items. Each item is rated on a 5-point Likert scale ranging from 1=thought never occurs to 5=thought always occurs; thus, a higher total score (range 14‐70) indicates more frequent or intense catastrophic thoughts [42]. Fear of bodily sensations was measured using the Body Sensations Questionnaire (BSQ), a 17-item self-report instrument capturing the fear and distress related to interoceptive sensations (eg, palpitations and dizziness) that often persist during panic symptoms. Each item is rated on a 5-point Likert scale from 1=not frightened or worried by this sensation to 5=extremely frightened; thus, a higher total score (range 17‐85) indicates greater fear of bodily sensations [43].

Adherence to the DTx during the 8-week intervention was objectively assessed using device use metrics. To evaluate adherence over time, the study period was divided into 3 phases: phase 1 (weeks 1‐2), phase 2 (weeks 3‐4), and phase 3 (weeks 5‐8). In each phase, successful adherence was defined as meeting the minimum prespecified protocol requirements. Specifically, participants were considered adherent for a given phase only if they met both of the following 2 conditions: first, completion of the training service modules allocated for that specific phase; and second, a record of using any module of the companion service or care service at least once within that phase. Participants failing to meet both criteria were classified as nonadherent for that phase. Finally, the total adherence rate for each participant was calculated as the percentage of successfully completed phases out of the total required phases ([number of adherent phases/3 total phases]×100). For cohort-level phase-specific adherence analyses, the denominators were adjusted to exclude participants with missing evaluations due to early withdrawal or discontinuation.

### Harms

Harms were assessed in both groups at every visit throughout the trial period. Participants were asked about any physical discomfort since the previous visit, and their reports were recorded on an adverse event worksheet. This worksheet allowed for checking the occurrence of physical discomforts during the study period; if reported, it was possible to record their details, including onset and end dates, range and severity of symptoms, causality, and the treatment and its results.

### Statistical Methods

All statistical analyses were performed using SAS (version 9.4; SAS Institute Inc) after data collection was completely finished, and interim analyses were not allowed. Baseline characteristics of participants were summarized descriptively; continuous variables were compared using a 2-tailed 2-sample *t* test (or Welch *t* test when variances were unequal), and categorical variables were compared using chi-square tests or exact tests when appropriate. For efficacy outcomes, between-group comparisons for changes from baseline in the primary and secondary end points were conducted using analysis of covariance (ANCOVA) adjusted for baseline scores, site, and sex. In addition to the absolute change, the percentage reduction from baseline in the primary outcome (PDSS-SR) was also analyzed between groups using ANCOVA adjusted for baseline scores, site, and sex. Responder rate was compared using Fisher exact test. All of these end points were analyzed based on the intention-to-treat approach. Missing data for both primary and secondary end points were handled using the last observation carried forward (LOCF) method. In addition, as part of the sensitivity analysis, a mixed model for repeated measures (MMRM) was conducted without imputation using all available observed data. All tests were 2-sided, and statistical significance was determined at an α level of .05. The effect size of Cohen *d* or odds ratio and the 95% CI were presented together. We did not adjust for multiple comparisons in the analysis of efficacy outcomes because the primary outcome variable was only the change in PDSS-SR scores at week 8; the changes in these scores at weeks 2 and 4 were exploratory analyses to evaluate the temporal trajectory of the treatment effect, and all analyses for secondary outcomes were intended to explore whether there would be an effect on other symptom aspects as well.

## Results

### Participant Flow

During the recruitment period, a total of 452 patients with panic disorder were evaluated for eligibility until the target number of 66 participants was competitively enrolled at the recruitment clinics of 6 institutions. To reach the final target sample size, the number of participants recruited at each institution varied from a minimum of 5 to a maximum of 19. These enrolled participants were randomly assigned to either the DTx (n=35) or control (n=31) group. One participant in the DTx group was excluded from the safety analysis because they did not receive the allocated intervention. Of these 65 participants, 4 participants (2 from each group) were excluded from the final efficacy analysis due to a lack of postbaseline efficacy assessments. Consequently, the final analysis included 61 participants (DTx: n=32; control: n=29; Figure 2), yielding an overall attrition rate of 7.57% (5/66), which was substantially lower than the anticipated 45% used in the sample size calculation.

**Figure 2.**
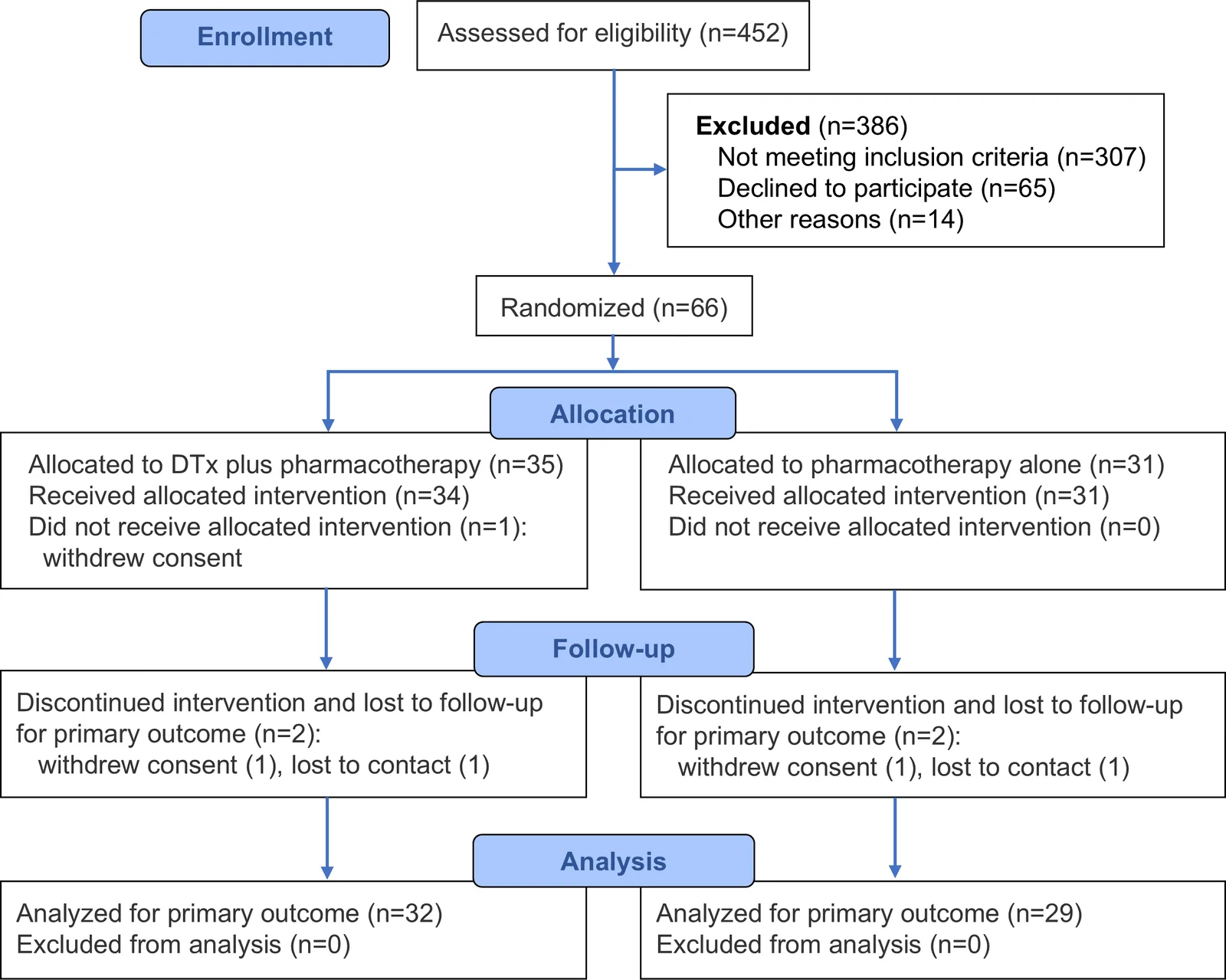
CONSORT (Consolidated Standards of Reporting Trials) flow diagram of participants in the digital therapeutic (DTx) and control groups (adapted from Hopewell et al [37], which is published under Creative Commons Attribution 4.0 International License [44]).

### Baseline Data and Missing Data

Baseline characteristics were generally balanced between groups (Table 1), including age (mean 35.31, SD 8.21 vs mean 34.69, SD 12.27 years), sex (male: 16/32, 50% vs 14/29, 48.28%), education level, psychiatric comorbidity, and the proportion of participants taking specific types of psychiatric medications, such as antidepressants, anxiolytics, hypnotics or sedatives, antipsychotics, and β-blocking agents (all *P*>.05).

**Table 1.** Demographic and clinical characteristics of participants at baseline in the digital therapeutic (DTx) and control groups^a^.

Characteristic	DTx (n=32)	Control (n=29)	Statistics (95% CI)	*P* value
Age (years), mean (SD)	35.31 (8.21)	34.69 (12.27)	Cohen *d*=0.06 (−0.44 to 0.56)	.82
Sex, n (%)			OR^b^=1.07 (0.39 to 2.93)	.89
Male	16 (50)	14 (48.28)		
Female	16 (50)	15 (51.72)		
Education level, n (%)			OR=0.55 (0.18 to 1.63)	.29
High school or less	8 (25)	11 (37.93)		
College or higher	24 (75)	18 (62.07)		
Psychiatric comorbidity, n (%)	9 (28.13)	12 (41.38)	OR=0.55 (0.19 to 1.61)	.41
Medication, n (%)				
Antidepressants	32 (100)	29 (100)	—^c^	—
Anxiolytics	30 (93.75)	27 (93.10)	OR=1.11 (0.15 to 8.44)	>.99
Hypnotics or sedatives	3 (9.38)	5 (17.24)	OR=0.50 (0.11 to 2.29)	.46
Antipsychotics	12 (37.50)	13 (44.83)	OR=0.74 (0.27 to 2.06)	.56
β-Blocking agents	13 (40.63)	10 (34.48)	OR=1.30 (0.46 to 3.68)	.62

a Age is compared using an independent 2-sample *t* test, with the effect size being Cohen *d*. The remaining variables are compared using a chi-square test (or Fisher exact test when appropriate), with the effect size being the odds ratio. Psychiatric comorbidity includes depressive disorders, anxiety disorders (eg, generalized anxiety disorder, social anxiety disorder, and agoraphobia), adjustment disorder, and others. Note that participants may have multiple diagnoses. Baseline concomitant medications were defined as psychiatric medications active at visit 1 (baseline), which included antidepressants (escitalopram, sertraline, paroxetine, fluoxetine, duloxetine, venlafaxine, desvenlafaxine, mirtazapine, vortioxetine, bupropion, agomelatine, amitriptyline, imipramine, milnacipran, tianeptine, and trazodone), anxiolytics (alprazolam, etizolam, lorazepam, clonazepam, and buspirone), hypnotics or sedatives (zolpidem, eszopiclone, zaleplon, and melatonin), antipsychotics (aripiprazole, quetiapine, olanzapine, risperidone, and lurasidone), and β-blocking agents (propranolol).

b OR: odds ratio.

c Not applicable.

Baseline PDSS-SR scores did not significantly differ between completers and noncompleters (mean 17.21, SD 3.95 and mean 19.33, SD 5.51, respectively; *P*=.38), supporting the assumption that data were missing completely at random. Among the 61 participants in the full analysis set, missing data followed a monotone dropout pattern at the participant level. There were no missing data at week 2, while data were missing at week 4 for 1 (1.61%) participant and at week 8 for 3 (4.92%) participants, as they were not evaluated. No item-level missing data were observed. In addition, the MMRM showed that the adjusted mean differences at weeks 2, 4, and 8 were −2.35 (95% CI −4.61 to −0.09; *P*=.04), −2.56 (95% CI −4.84 to −0.29; *P*=.03), and −3.97 (95% CI −6.27 to −1.68; *P*<.001), respectively, which were consistent in both direction and magnitude compared to the LOCF-based analysis results of −2.24 (95% CI −4.00 to −0.48; *P*=.02), −2.54 (95% CI −4.85 to −0.23; *P*=.04), and −3.77 (95% CI −6.36 to −1.18; *P*=.006). The CIs from the 2 approaches overlapped substantially at every time point, confirming that the primary findings were robust to the method used to handle missing data.

### Concomitant Care

Both groups of patients received conventional pharmacotherapy during the trial period. Regarding psychiatric medications, results showing no significant difference in the number of participants in each group who took a specific type of medication are presented in Table S1 in Multimedia Appendix 1. Detailed results regarding medication adjustments are presented in Table S2 in Multimedia Appendix 1, which lists all psychiatric medications used and provides specific figures for each medication, including the number of users, dosage range, duration of use, and dosage changes. Tables S3 and S4 are also added to Multimedia Appendix 1 to compare the 2 groups regarding regimen adjustments and dosage changes during the trial period for each type of psychiatric medication, respectively. As shown in these tables, no type of psychiatric medication showed significant group differences in use duration and dosage changes. Overall, the proportion of participants whose medication regimen was adjusted through new start, discontinuation, irregular, or reuse over the trial period was similar between groups (DTx: 18/32, 56.25%; control: 17/29, 58.62%; odds ratio 0.91, 95% CI 0.33-2.51; *P*>.99), and the proportion whose dose changed was also similar (DTx: 27/32, 84.37%; control: 23/29, 79.31%; odds ratio 1.41, 95% CI 0.38-5.22; *P*=.74).

### Primary Outcome

The change in PDSS-SR scores, the primary outcome variable, is shown in Table 2. The mean baseline PDSS-SR scores were similar between the DTx and control groups (mean 17.22, SD 3.87 vs mean 17.41, SD 4.23; Cohen *d*=−0.05, 95% CI −0.55 to 0.46; *P*=.85). At week 2, the mean PDSS-SR score decreased to 12.81 (SD 5.13) in the DTx group and to 15.52 (SD 5.17) in the control group; percentage reduction from baseline was significantly greater in the DTx group than in the control group (mean 26.66%, SD 22.03% vs mean 11.05%, SD 21.73%; Cohen *d*=0.71, 95% CI 0.19-1.23; *P*=.02). At week 4, the mean score further decreased to 11.97 (SD 5.74) in the DTx group and 14.83 (SD 6.76) in the control group. Percentage reduction from baseline increased further in both groups and showed a greater trend in the DTx group than in the control group (mean 31.13%, SD 27.01% vs mean 16.42%, SD 29.90%; Cohen *d*=0.52, 95% CI 0.01-1.03; *P*=.05). At week 8, the mean score decreased further in both groups (mean 10.31, SD 5.13 and mean 14.38, SD 6.51, respectively), and percentage reduction was significantly greater in the DTx group than in the control group (mean 40.29%, SD 26.68% vs mean 17.61%, SD 30.37%; Cohen *d*=0.80, 95% CI 0.28-1.32; *P*=.007; Figure 3). In addition, the responder rate (defined as a ≥40% PDSS-SR reduction at week 8 with LOCF) was significantly higher in the DTx group compared to the control group (17/32, 53.12% vs 5/29, 17.24%; odds ratio 5.44, 95% CI 1.66-17.84; *P*=.007). The corresponding absolute risk reduction was 35.88% (95% CI 13.79%-57.97%), yielding a number needed to treat of 2.8 (95% CI 1.7-7.2).

**Table 2.** Primary outcome: changes in Panic Disorder Severity Scale-Self Report (PDSS-SR) scores over 8 weeks in the digital therapeutic (DTx) and control groups^a^.

Variable	DTx (n=32)	Control (n=29)	Adjusted mean difference (95% CI)	Statistics (95% CI)	*P* value
Baseline, mean (SD)	17.22 (3.87)	17.41 (4.23)	—^b^	Cohen *d*=−0.05 (−0.55 to 0.46)	.85
Change from baseline, mean (SD)					
Week 2	−4.41 (3.56)	−1.90 (3.54)	−2.24 (−4.00 to −0.48)	Cohen *d*=0.71 (0.19 to 1.23)	.02
Week 4	−5.25 (4.79)	−2.59 (4.98)	−2.54 (−4.85 to −0.23)	Cohen *d*=0.54 (0.03 to 1.05)	.04
Week 8	−6.91 (4.70)	−3.03 (5.45)	−3.77 (−6.36 to −1.18)	Cohen *d*=0.77 (0.25 to 1.29)	.006
Percent reduction from baseline, mean (SD)					
Week 2	26.66 (22.03)	11.05 (21.73)	13.51 (2.58 to 24.44)	Cohen *d*=0.71 (0.19 to 1.23)	.02
Week 4	31.13 (27.01)	16.42 (29.90)	13.48 (−0.28 to 27.24)	Cohen *d*=0.52 (0.01 to 1.03)	.06
Week 8	40.29 (26.68)	17.61 (30.37)	21.39 (6.22 to 36.57)	Cohen *d*=0.80 (0.28 to 1.32)	.007
Responder analysis, n (%)					
Responder	17 (53.12)	5 (17.24)	—	OR^c^=5.44 (1.66 to 17.84)	.007
Nonresponder	15 (46.88)	24 (82.76)	—	—	—

a Baseline scores are compared using an independent 2-sample *t* test, with the effect size being Cohen *d*. In change and percent reduction from baseline, adjusted mean differences and *P* values were derived from analysis of covariance adjusted for baseline scores, site, and sex. Responder was defined as a ≥40% reduction in the PDSS-SR total score from baseline to week 8, and responder rate was compared using Fisher exact test, with the effect size being the OR.

b Not applicable.

c OR: odds ratio.

**Figure 3.**
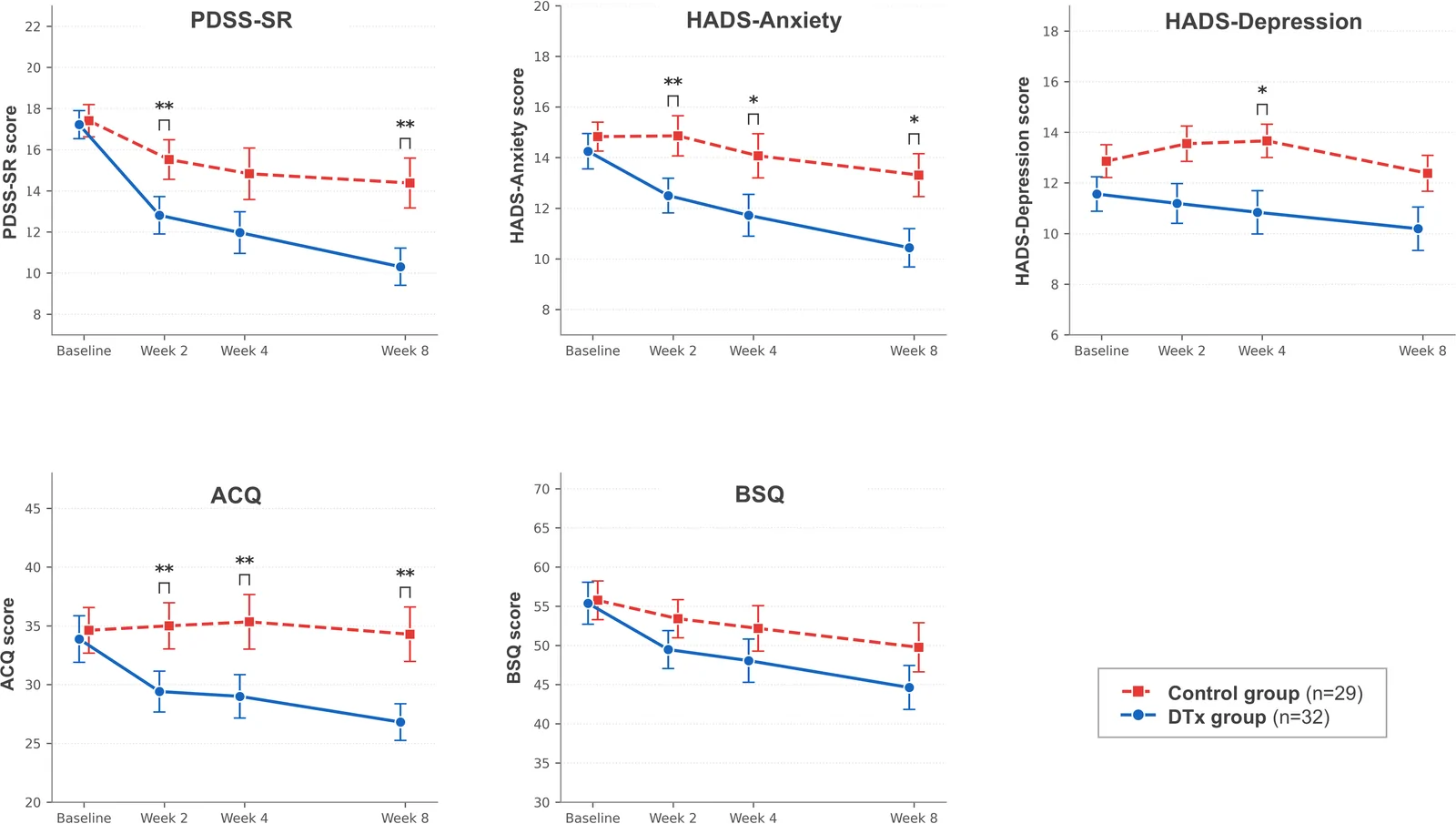
Changes in primary and secondary outcome scores over 8 weeks in the digital therapeutic (DTx) and control groups. Values are mean (SE). The primary outcome was measured using the Panic Disorder Severity Scale-Self Report (PDSS-SR), and secondary outcomes were scored using the Hospital Anxiety and Depression Scale (HADS), Agoraphobic Cognitions Questionnaire (ACQ), and Body Sensations Questionnaire (BSQ). Between-group comparisons were performed using analysis of covariance adjusted for baseline scores, site, and sex. *P<.05, **P<.01.

### Secondary Outcomes

Secondary outcomes, including the HADS, ACQ, and BSQ, are summarized in Table 3. As shown in Figure 3, HADS-Anxiety scores were significantly reduced in the DTx group than in the control group at weeks 2, 4, and 8 (Cohen *d*=0.77, 95% CI 0.25-1.29; *P*=.005; Cohen *d*=0.53, 95% CI 0.02-1.04; *P*=.03; and Cohen *d*=0.65, 95% CI 0.13-1.17; *P*=.01, respectively). For HADS-Depression scores, the DTx group demonstrated a significantly greater reduction at week 4 (Cohen *d*=0.44, 95% CI −0.07 to 0.95; *P*=.04), but the between-group differences at weeks 2 and 8 were not statistically significant. ACQ scores showed significantly greater improvement in the DTx group compared to the control group at weeks 2, 4, and 8 (Cohen *d*=0.73, 95% CI 0.21-1.25; *P*=.003; Cohen *d*=0.71, 95% CI 0.19-1.23; *P*=.004; and Cohen *d*=0.88, 95% CI 0.35-1.41; *P*=.001, respectively). Changes in BSQ scores did not differ significantly between groups at weeks 2, 4, or 8.

**Table 3.** Secondary outcomes: changes in anxiety, depression, agoraphobic cognitions, and body sensations over 8 weeks in the digital therapeutic (DTx) and control groups^a^.

Measures	DTx (n=32), mean (SD)	Control (n=29), mean (SD)	Adjusted mean difference (95% CI)	Cohen *d* (95% CI)	*P* value
HADS-Anxiety^b^					
Baseline score	14.25 (3.95)	14.83 (3.08)	—^c^	−0.16 (−0.67 to 0.34)	.53
Change at week 2	−1.75 (2.33)	0.03 (2.34)	−1.83 (−3.09 to −0.57)	0.77 (0.25 to 1.29)	.005
Change at week 4	−2.53 (3.15)	−0.76 (3.57)	−1.82 (−3.45 to −0.20)	0.53 (0.02 to 1.04)	.03
Change at week 8	−3.81 (3.32)	−1.52 (3.73)	−2.34 (−4.09 to −0.59)	0.65 (0.13 to 1.17)	.01
HADS-Depression					
Baseline score	11.56 (3.84)	12.86 (3.49)	—	−0.35 (−0.86 to 0.15)	.17
Change at week 2	−0.38 (2.18)	0.69 (2.47)	−1.11 (−2.35 to 0.12)	0.46 (−0.05 to 0.97)	.08
Change at week 4	−0.72 (3.30)	0.79 (3.58)	−1.88 (−3.65 to −0.10)	0.44 (−0.07 to 0.95)	.04
Change at week 8	−1.38 (3.68)	−0.48 (3.65)	−1.38 (−3.27 to 0.51)	0.24 (−0.26 to 0.74)	.15
ACQ^d^					
Baseline score	33.88 (11.20)	34.62 (10.46)	—	−0.07 (−0.57 to 0.43)	.79
Change at week 2	−4.47 (5.22)	0.38 (7.90)	−4.90 (−7.94 to −1.86)	0.73 (0.21 to 1.25)	.003
Change at week 4	−4.88 (6.26)	0.72 (9.28)	−5.63 (−9.26 to −2.00)	0.71 (0.19 to 1.23)	.004
Change at week 8	−7.06 (5.62)	−0.34 (9.36)	−6.76 (−10.25 to −3.27)	0.88 (0.35 to 1.41)	.001
BSQ^e^					
Baseline score	55.38 (15.19)	55.76 (13.29)	—	−0.03 (−0.53 to 0.48)	.92
Change at week 2	−5.91 (10.46)	−2.34 (8.31)	−3.68 (−8.72 to 1.35)	0.38 (−0.13 to −0.89)	.15
Change at week 4	−7.31 (9.46)	−3.59 (9.47)	−3.61 (−8.37 to 1.15)	0.39 (−0.12 to −0.90)	.13
Change at week 8	−10.75 (8.89)	−6.00 (9.96)	4.81 (−10.31 to 0.70)	0.50 (−0.01 to 1.01)	.09

a Data are compared using an independent 2-sample *t* test, with the effect size being Cohen *d*. Adjusted mean differences and *P* values were derived from analysis of covariance adjusted for baseline scores, site, and sex.

b HADS: Hospital Anxiety and Depression Scale.

c Not applicable.

d ACQ: Agoraphobic Cognitions Questionnaire.

e BSQ: Body Sensations Questionnaire.

### Adherence

The results of adherence are presented in Table 4. The overall mean total adherence rate was 78.12% (SD 34.51%), with no significant difference between responders and nonresponders. Successful adherence rates by phase were as follows: weeks 1‐2: 90.62% (29/32), weeks 3‐4: 77.42% (24/31), and weeks 5‐8: 63.33% (19/30), with no significant differences based on responder status at any phase. Successful adherence rates by content level (training and companion or care) did not also differ significantly between responders and nonresponders.

**Table 4.** Device adherence rate in the digital therapeutic group^a^.

Variable	Total (N=32)	Responder (n=17)	Nonresponder (n=15)	Statistics (95% CI)	*P* value
Total adherence rate, mean (SD)	78.12 (34.51)	76.47 (36.83)	80.00 (32.85)	Cohen *d*=−0.10 (−0.80 to 0.59)	.86
Adherence success, n (%)					
Weeks 1‐2	29 (90.62)	15 (88.24)	14 (93.33)	OR^b^=0.54 (0.04 to 6.58)	>.99
Weeks 3‐4	24 (77.42)	13 (76.47)	11 (78.57)	OR=0.89 (0.16 to 4.85)	>.99
Weeks 5‐8	19 (63.33)	11 (64.71)	8 (61.54)	OR=1.15 (0.26 to 5.11)	.72
Training, n (%)					
Weeks 1‐2	29 (90.62)	15 (88.24)	14 (93.33)	OR=0.54 (0.04 to 6.58)	>.99
Weeks 3‐4	24 (77.42)	13 (76.47)	11 (78.57)	OR=0.89 (0.16 to 4.85)	>.99
Weeks 5‐8	21 (70)	12 (70.59)	9 (69.23)	OR=1.07 (0.22 to 5.14)	.71
Companion or care, n (%)					
Weeks 1‐2	32 (100)	17 (100)	15 (100)	—^c^	>.99
Weeks 3‐4	31 (100)	17 (100)	14 (100)	—	>.99
Weeks 5‐8	27 (90)	16 (94.12)	11 (84.62)	OR=2.91 (0.23 to 36.17)	.32

a Total adherence rate was calculated as (number of compliant phases/total phases)×100, which was compared using the Mann-Whitney *U* test, with the effect size being Cohen *d*. The remaining variables were compared using the Fisher exact test, with the effect size being the OR. Adherence success was defined as meeting both of the following conditions within each phase: (1) completing the training service modules allocated for that phase and (2) having a record of using any companion or care service module at least once. Training and companion or care rows represent participants who met each individual criterion. Denominators may vary due to missing assessments or discontinuation.

b OR: odds ratio.

c Not applicable.

### Harms

No clinically significant adverse device effects were identified in the DTx group during the 8-week study period. In total, 7 patients in the DTx group reported a total of 12 physical discomforts, including a cold, heartburn, shoulder pain, tongue pain, hematuria, and elevated liver enzyme levels; all of these were assessed as unrelated to or unlikely related to the DTx. Meanwhile, 2 patients in the control group also reported 2 physical discomforts, such as a cold and a hand fracture.

## Discussion

This multicenter randomized controlled trial evaluated the efficacy of a prescription-based DTx as an adjunct to pharmacotherapy for acute-phase panic disorder. Changes in overall panic symptom severity for the primary objective of the efficacy evaluation were significantly greater in the DTx group than in the control group. More than half of the participants in the DTx group achieved a clinically meaningful response in overall panic symptom severity, which was nearly 3 times higher than the control group. In the evaluation of the secondary objectives—changes in overall anxiety and depression, panic-related catastrophic cognitions, and the fear of bodily sensations—different results were observed by domain; the DTx group showed significant improvement in overall anxiety and catastrophic cognitions compared to the control group, but such improvement was not observed in depression and the fear of bodily sensations.

The most obvious result regarding the improvement of panic symptoms is that at the primary end point of week 8, the DTx group showed significantly superior results in the reduction of PDSS-SR scores compared to the control group, suggesting that the adjunctive use of DTx provides additional benefits in symptom improvement compared with pharmacotherapy alone. Notably, this therapeutic effect was already observed in week 2, indicating that the benefits of the DTx are evident from the early stage. Beyond panic symptom severity, the clinical benefits of the DTx extended to overall anxiety and panic-related catastrophic cognitions. The DTx group demonstrated significantly greater improvements in these measures at all postbaseline visits, suggesting that the DTx effectively addresses both the core panic symptoms and the broad cognitive-emotional factors that perpetuate the disorder. Taken together, the early-onset efficacy and comprehensive improvement across various symptom domains underscore the potential of this DTx as an efficient tool for managing panic disorder in real-world clinical settings.

Meanwhile, when discussing the effectiveness of DTx use, we must consider that since sham use was not included in the control group, it is difficult to rule out the possibility that the potential influence of expectancy effects of digital participation using the new device or the additional effects of nonspecific treatment factors including increased clinical attention may have been involved in the superior effect in the DTx group. In this regard, we need to discuss the magnitude of the effectiveness. The magnitude of symptom reduction and responder rates observed in this study aligns with the established efficacy of both digital interventions and conventional therapies for panic disorder. Previous meta-analyses evaluating internet-based CBT and mobile health interventions for anxiety have reported comparable symptom reduction and responder rates, typically ranging between 40% and 55% [45-47]. Notably, the effect size observed for the primary outcome in our study (Cohen *d*=0.80) represents a large effect, which is comparable to or exceeding those reported for clinician-guided digital interventions for panic disorder (Hedges *g*=0.95) and substantially larger than self-guided formats (Hedges *g*=0.31) in a recent meta-analysis [24]. Furthermore, the 17.61% symptom reduction and 17.24% (5/29) responder rate observed in our control group are very similar to the modest short-term trajectories reported in previous meta-analyses for treatment-as-usual or placebo conditions in panic disorder trials [39,48]. This consistency shown in the control group may confirm that the group served as a valid comparator representing conventional care, and the unsatisfactory treatment response further highlights the unmet clinical need for adjunctive interventions to address acute panic symptoms.

A challenge for many existing self-directed mobile health apps has been high attrition rates and rapidly declining user engagement, which frequently dilute their clinical impact outside of strictly controlled clinical trials [49]. Our findings demonstrated high patient adherence, achieving high success rates not only in the initial phase but also overall, suggesting that this challenge may be relatively less problematic. We believe that this high level of adherence may be driven by several factors. This was likely facilitated not only by the app’s comprehensive, user-centered design integrating structured CBT training, daily lifestyle self-management, and real-time symptom management support, but also by its prescription-based framework. Rather than relying solely on patient self-motivation, this synergistic approach combines a well-designed digital tool with active integration into the psychiatric treatment workflow. Knowing that their attending clinician monitors their progress via a web-based console likely can make patients feel a sense of accountability in them and strengthen the therapeutic alliance [50,51]. This “clinician-in-the-loop” oversight effectively mitigates the “digital fatigue” commonly reported in prior literature, ensuring that patients consistently engage with the intervention [52]. Although we observed a gradual decrease in phase-specific adherence over time, this pattern is typical in prolonged digital health interventions. Meanwhile, given that there was no significant difference in adherence between responders and nonresponders, high adherence cannot be seen as directly related to therapeutic efficacy, nor can the decrease in adherence over time be attributed to a natural reduction in the need for daily app use following rapid symptom alleviation. However, in making this interpretation, it should be considered that the statistical power of the subgroup analysis regarding the difference in adherence between the 2 groups may have been limited due to the relatively small sample size of this study.

The early and sustained reduction in symptoms, particularly the significant between-group differences observed as early as week 2, suggests that specific therapeutic mechanisms may be active during the initial phase of the intervention. Traditional face-to-face CBT often encounters challenges with skill generalization, as patients frequently struggle to recall and apply coping strategies during unexpected panic episodes in real-life situations [53]. The intervention in the current study appears to mitigate this practical difficulty through the companion service, which delivers JITAIs. By providing immediate, context-responsive tools, such as paced breathing and grounding techniques, at the moment of physiological arousal, the app assists patients in managing their symptoms before they worsen. This real-time intervention appears to have been particularly relevant to rapid improvements in ACQ scores. Catastrophic cognitions in panic disorder tend to persist when autonomic arousal is misinterpreted as an imminent physical or mental threat [54]. Delivering JITAIs via the companion service during these acute moments has the potential to reduce the patients’ feared catastrophic consequences of physiological arousal and to lead to an experience of in vivo cognitive restructuring. This potential experiential learning is more likely to disrupt the “fear-of-fear” cycle more rapidly than delayed reflection in a clinic setting.

Beyond the management of acute panic symptoms, the significant reduction in overall anxiety levels (HADS-Anxiety) observed throughout the 8-week period likely reflects the comprehensive nature of the DTx, particularly the synergy between cognitive-behavioral skill acquisition and daily self-management. While the training and companion services directly target cognitive distortions and acute arousal associated with anticipatory anxiety, the care service addresses underlying physiological vulnerability. Panic disorder is frequently exacerbated by lifestyle factors, such as irregular sleep patterns or excessive caffeine consumption, which heighten baseline physiological arousal [17,55]. By systematically identifying and modifying these daily habits alongside structured CBT practice, patients can actively lower their everyday physiological vulnerability [56]. This continuous, multifaceted self-regulation likely contributed to a steady decline in overall anxiety, providing a stabilizing effect between clinical visits.

While the intervention demonstrated consistent effects on the severity of panic symptoms, overall anxiety, and catastrophic cognitions, changes in other secondary domains, such as depression or fear of bodily sensations, were transient or insignificant, suggesting that its effects may be confined to specific core domains rather than being general. A significant difference in HADS-Depression scores between the DTx and control groups was observed only at week 4, but it is difficult to view this as a result reflecting the therapeutic effect of the DTx, because it appears to be due to a temporary worsening in the control group rather than improvement in the DTx group. The intermittent significance in these scores may reflect the secondary and highly variable nature of mood symptoms in this population. Mild-to-moderate depression often arises as a secondary reaction to psychosocial impairment caused by unpredictable panic attacks, and thus may improve or worsen depending on the immediate severity of panic behaviors [3]. However, in cases of severe depression, recovery from depressive symptoms may be slow even if panic symptoms improve first [57]. Therefore, the 8-week time frame of using DTx seems insufficient to capture stable, long-term modifications in these comorbid mood symptoms. Meanwhile, the lack of significant between-group differences in BSQ scores at all measurement points may reflect the nature of the self-guided approach of the DTx. A previous study reporting improvements in this scale in patients with panic disorder observed the effectiveness of cognitive therapy conducted through a sufficient number of face-to-face sessions with a therapist [58]. Fear of bodily sensations measured by this scale is typically addressed through interoceptive exposure, which often requires repetitive and high-intensity symptom provocation [43]. However, the self-guided approach in a real-world environment may not have allowed for sufficient intensity or dosage of interoceptive exposure required to significantly alter these specific somatic fears. These findings suggest that while the DTx effectively manages acute panic and cognitive appraisals, certain symptom domains may require extended treatment durations or more targeted, therapist-guided exposure components.

While pharmacotherapy is the cornerstone of standard treatment for panic disorder, many patients continue to experience persistent panic symptoms and their aftereffects, particularly anticipatory anxiety and avoidance behaviors, despite such treatment [59]. Our results indicate that the adjunctive use of the DTx provides meaningful additional symptom reduction to an extent that cannot be achieved with pharmacotherapy alone. Notably, these benefits were observed in a naturalistic setting where clinicians adjusted medication regimens according to clinical needs. Because this approach closely mirrors routine outpatient care, the generalizability of our findings can be extended to everyday psychiatric practice. By using a prescription-based digital tool for patients coupled with a monitoring dashboard for clinicians, psychiatrists can provide evidence-based behavioral interventions while efficiently managing patient progress, even within the time constraints of outpatient visits. Furthermore, the observed attrition rate was markedly lower than anticipated during study planning, further supporting the feasibility and acceptability of this multifunctional DTx. Despite integrating multiple therapeutic components within a single protocol, participant retention remained high, suggesting that the comprehensive design did not impose an excessive burden but rather facilitated sustained engagement throughout the intervention period. It must be noted here that the observed low attrition rate may be associated with a selected sample of voluntary participants in the controlled environment of a clinical trial. Therefore, it is uncertain whether this result will be actually maintained in routine clinical practice; thus, verification in real-world clinical settings will be necessary.

Several limitations should be considered when interpreting the results of this study. First, the primary and secondary efficacy end points relied on self-report questionnaires to assess the symptom severity subjectively perceived by patients as an indicator of effectiveness. While these questionnaires are widely validated and used as standards in panic disorder research, they are inherently susceptible to reporting bias. In addition, no adjustment was made for multiple comparisons in analyzing these efficacy end points, as the analyses for variables other than the single primary end-point variable were entirely exploratory; thus, it should be noted that the interpretation of the results cannot be definitive due to the increased risk of type I error. Second, the control group received pharmacotherapy alone without a sham digital intervention, despite consenting to participate in the study. Consequently, in addition to the aforementioned issue regarding the expectancy effects of digital participation and additional nonspecific therapeutic elements in the DTx group, it is possible that the inability to use a sham device also affected the control group in another way. That is, it cannot be entirely ruled out that not only did they fail to obtain a placebo effect from sham use, but the disappointment stemming from their inability to use a novel device also had a negative impact on the symptom assessment. Third, although medication adjustments were permitted based on symptom status at the discretion of clinicians who were unaware of the participants’ group assignments, some potential pharmacological confounding factors may not have been controlled. A post hoc review of concomitant medication records indicated that use duration and dosage changes for all types of psychiatric medications were similar between the 2 groups. However, it was not possible to determine whether there were differences in pharmacological intensity between the groups due to the difficulty in quantifying dosages by type, nor could it be confirmed whether there was any biased use toward specific medications, given the small number of users for each medication due to the use of various ones. Finally, the 8-week trial duration captures only the acute treatment phase. It remains unclear whether the observed symptom reductions achieved through a digital platform and high patient engagement persist over a longer follow-up period, or if symptoms worsen again after the formal intervention. This needs to be clarified through a future study including a posttrial follow-up of 3 or 6 months.

In conclusion, this multicenter randomized controlled trial indicates that our prescription-based DTx is a feasible adjunct to pharmacotherapy for panic disorder, demonstrating particular clinical value for acute-phase patients experiencing frequent panic attacks. The adjunctive use of the DTx resulted in early and sustained symptom reductions across various domains, including panic severity, overall anxiety, and catastrophic cognitions, and demonstrated a clinically meaningful responder rate. This self-guided intervention, which integrates structured CBT, just-in-time context-responsive coping tools, and daily lifestyle tracking, supported sustained patient adherence throughout the 8-week period. Taken together, the results of the current study suggest that this integrated DTx model can serve as a practical complement to routine psychiatric care for patients experiencing frequent panic attacks. Furthermore, this model is expected to present a new framework for establishing a new doctor-patient relationship in the treatment of acute-phase panic disorder by reflecting various aspects related to symptoms manifesting in the patient’s daily life into the clinician’s evaluation and guidance in the clinic. Future large-scale studies with extended follow-up periods are warranted to further establish the long-term utility of this intervention.

## Supplementary material

10.2196/101125 Multimedia Appendix 1 Supplementary tables.

10.2196/101125 Checklist 1 CONSORT-eHEALTH checklist (V 1.6.1).

10.2196/101125 Checklist 2 CONSORT 2025 checklist.

## Abbreviations

ACQ: Agoraphobic Cognitions Questionnaire
ANCOVA: analysis of covariance
BSQ: Body Sensations Questionnaire
CBT: cognitive behavioral therapy
CONSORT-eHEALTH: Consolidated Standards of Reporting Trials of Electronic and Mobile Health Applications and Online Telehealth
*DSM-5*: *Diagnostic and Statistical Manual of Mental Disorders, Fifth Edition*
DTx: digital therapeutic
HADS: Hospital Anxiety and Depression Scale
*ICD-10*: *International Classification of Diseases and Related Health Problems, Tenth Edition*
JITAI: just-in-time adaptive intervention
LOCF: last observation carried forward
MMRM: mixed model for repeated measures
PDSS-SR: Panic Disorder Severity Scale-Self Report
SSRI: selective serotonin reuptake inhibitor
