# Supplementary material for: Efficacy of a Prescription-Based Mobile Digital Therapeutic as an Adjunct to Pharmacotherapy for the Acute Phase of Panic Disorder: Multicenter Randomized Controlled Trial
Source: J Med Internet Res. 2026 Oct 5;28:e101125. doi: 10.2196/101125 (PMC13637997; doi:10.2196/101125)
Supplement: Supplementary file 1 [file jmir-v28-e101125-s001.pdf]

Supplementary Tables

Table S1. The number (percentage) of participants who took the specific type of medication during the trial period. Comparison between groups was based on the Fisher's exact test with the odds ratio to determine whether the specific type of medication was administered.

Characteristic	DTx (N=32)	Control (N=29)	Odds Ratio (95% CI)	*P*
Antidepressants	32 (100.00)	29 (100.00)	—	>.99
Anxiolytics	32 (100.00)	28 (96.55)	—	.48
Hypnotics/Sedatives	4 (12.50)	5 (17.24)	0.69 (0.17 to 2.85)	.72
Antipsychotics	16 (50.00)	16 (55.17)	0.81 (0.30 to 2.23)	.80
Beta-blocking agents	15 (46.88)	15 (51.72)	0.82 (0.30 to 2.25)	.80

Note: Antidepressants included escitalopram, sertraline, paroxetine, fluoxetine, duloxetine, venlafaxine, desvenlafaxine, mirtazapine, vortioxetine, bupropion, agomelatine, amitriptyline, imipramine, milnacipran, tianeptine, and trazodone. Anxiolytics included alprazolam, etizolam, lorazepam, clonazepam, diazepam and buspirone. Hypnotics/sedatives included zolpidem, eszopiclone, zaleplon, melatonin and doxepin. Antipsychotics included aripiprazole, quetiapine, olanzapine, risperidone, and lurasidone. Beta-blocking agents included propranolol. Abbreviations: CI, confidence interval.

Table S2. Number of participants using psychiatric medications, daily dosage range, duration of administration, and whether dosage changed during the study period in the digital therapeutic (DTx) and control groups.

Measures	DTx (N=32)				Control (N=29)			
	Users	Dosage range, mg	Duration (Co./Ne./Di./Ir./Re.)	Dosage changes (Un./In./De.)	Users	Dosage range, mg	Duration (Co./Ne./Di./Ir./Re.)	Dosage changes (Un./In./De.)
**Antidepressants**								
Escitalopram	20	5-20	16/1/1/1/1	14/6/0	19	5-20	12/3/3/1/0	9/4/6
Sertraline	7	50-200	7/0/0/0/0	4/2/1	6	25-200	2/3/1/0/0	4/2/0
Paroxetine	3	25-50	2/0/1/0/0	2/0/1	1	75	1/0/0/0/0	1/0/0
Fluoxetine	1	20-40	1/0/0/0/0	0/1/0	3	20-40	3/0/0/0/0	3/0/0
Duloxetine	1	30-60	1/0/0/0/0	0/0/1	1	60	1/0/0/0/0	1/0/0
Venlafaxine	1	75	1/0/0/0/0	1/0/0	2	37.5-150	1/1/0/0/0	2/0/0
Desvenlafaxine	3	50-200	2/1/0/0/0	2/1/0	6	50-200	4/0/1/1/0	5/1/0
Mirtazapine	5	3.75-30	4/1/0/0/0	3/1/1	9	3.75-30	6/1/1/1/0	6/2/1
Vortioxetine	2	5-10	1/0/0/0/1	1/1/0	5	5-10	2/1/2/0/0	3/1/1
Bupropion	2	150-300	2/0/0/0/0	1/1/0	2	150-300	1/0/0/1/0	1/1/0
Agomelatine	4	25-50	3/1/0/0/0	3/1/0				
Amitriptyline					2	5-10	1/0/1/0/0	2/0/0
Imipramine					1	25	1/0/0/0/0	1/0/0
Milnacipran					1	12.5-50	1/0/0/0/0	0/1/0
Tianeptine					1	25	1/0/0/0/0	1/0/0
Trazodone	5	12.5-100	3/2/0/0/0	4/0/1	8	25-75	4/3/0/1/0	8/0/0
**Anxiolytics**								
Alprazolam	31	0.125-1.5	22/1/4/3/1	19/2/10	26	0.0625-1.5	20/1/2/2/1	16/3/7
Etizolam	4	0.25-3	2/0/1/0/1	3/0/1	4	0.125-1.5	0/2/1/1/0	3/0/1
Lorazepam	6	0.25-3	2/2/1/1/0	5/1/0	8	0.25-2	5/2/1/0/0	5/2/1
Clonazepam	11	0.25-1.5	6/3/2/0/0	11/0/0	15	0.25-1.5	9/4/1/1/0	11/3/1
Diazepam	1	2	0/1/0/0/0	1/0/0	1	2-5	0/1/0/0/0	1/0/0
Buspirone					2	10-30	1/0/0/1/0	2/0/0
**Hypnotics/Sedatives**								
Zolpidem					1	10	1/0/0/0/0	1/0/0
Eszopiclone					2	1-2	1/0/1/0/0	2/0/0
Zaleplon	3	5-10	2/1/0/0/0	2/0/1				
Melatonin	1	2	1/0/0/0/0	1/0/0	2	2	2/0/0/0/0	2/0/0
Doxepin	1	6	1/0/0/0/0	1/0/0	2	6	2/0/0/0/0	2/0/0
**Antipsychotics**								
Aripiprazole	7	1-5	5/2/0/0/0	5/2/0	9	1-5	5/2/1/0/1	6/3/0
Quetiapine	9	12.5-200	7/1/1/0/0	6/3/0	12	6.25-100	5/4/2/1/0	8/1/3
Olanzapine	1	2.5	1/0/0/0/0	1/0/0	3	1.25-10	1/2/0/0/0	1/0/2
Risperidone	1	2-3	1/0/0/0/0	0/0/1				
Lurasidone					1	80	1/0/0/0/0	1/0/0
**Beta-blocking agents**								
Propranolol	15	10-60	14/1/0/0/0	12/1/2	15	10-90	10/5/0/0/0	14/0/1

Note: Users indicate the number of participants who took the medicine. Dosage range indicates the actual prescribed daily dose range of the medicine. In duration, continuous use (Co.), new start (Ne.), discontinuation (Di.), irregular use (Ir.), and reuse (Re.) represent the number of participants who took the medicine throughout the study period, started taking it midway, stopped taking it midway, took it intermittently as needed, and stopped taking it midway and then resumed, respectively. In dosage changes, Un., In., and De. represent the number of participants whose dose of the medicine remained unchanged, increased, and decreased, respectively, during the study period.

Table S3. The number of participants whose medication regimen was continuously maintained or adjusted (including new start, discontinuation, irregular use, and reuse) during the trial period. The comparison between groups regarding whether the type of medication was adjusted was based on Fisher's exact test with the odds ratio.

Characteristic	DTX (N=32)	Control (N=29)	Odds Ratio (95% CI)	*P*
Any psychiatric medication	32	29		
Maintained	14 (43.75)	12 (41.38)		
Adjusted	18 (56.25)	17 (58.62)	0.91 (0.33 to 2.51)	>.99
Antidepressants	32	29		
Maintained	24 (75.00)	18 (62.07)		
Adjusted	8 (25.00)	11 (37.93)	0.55 (0.18 to 1.63)	.41
Anxiolytics	32	28		
Maintained	18 (56.25)	17 (60.71)		
Adjusted	14 (43.75)	11 (39.29)	1.20 (0.43 to 3.37)	.80
Hypnotics/Sedatives	4	5		
Maintained	3 (75.00)	4 (80.00)		
Adjusted	1 (25.00)	1 (20.00)	1.33 (0.06 to 31.12)	>.99
Antipsychotics	16	16		
Maintained	12 (75.00)	7 (43.75)		
Adjusted	4 (25.00)	9 (56.25)	0.26 (0.06 to 1.16)	.15
Beta-blocking agents	15	15		
Maintained	14 (93.33)	10 (66.67)		
Adjusted	1 (6.67)	5 (33.33)	0.14 (0.01 to 1.42)	.17

Note: Antidepressants included escitalopram, sertraline, paroxetine, fluoxetine, duloxetine, venlafaxine, desvenlafaxine, mirtazapine, vortioxetine, bupropion, agomelatine, amitriptyline, imipramine, milnacipran, tianeptine, and trazodone. Anxiolytics included alprazolam, etizolam, lorazepam, clonazepam, diazepam and buspirone. Hypnotics/sedatives included zolpidem, eszopiclone, zaleplon, melatonin and doxepin. Antipsychotics included aripiprazole, quetiapine, olanzapine, risperidone, and lurasidone. Beta-blocking agents included propranolol. Abbreviations: CI, confidence interval.

Table S4. The number (percentage) of participants whose psychiatric medication dosage was unchanged and changed (increased or decreased) during the trial period. The comparison between groups regarding whether the dose of medication was changed was based on Fisher's exact test with the odds ratio.

**Characteristic**	**DTX (N=32)**	**Control (N=29)**	**Odds Ratio (95% CI)**	***P***
Any psychiatric medication	32	29		
Unchanged	5 (15.62)	6 (20.69)		
Changed	27 (84.38)	23 (79.31)	1.41 (0.38 to 5.22)	.74
Antidepressants	32	29		
Unchanged	15 (46.88)	13 (44.83)		
Changed	17 (53.12)	16 (55.17)	0.92 (0.34 to 2.53)	>.99
Anxiolytics	32	28		
Unchanged	18 (56.25)	14 (50.00)		
Changed	14 (43.75)	14 (50.00)	0.78 (0.28 to 2.15)	.80
Hypnotics/Sedatives	4	5		
Unchanged	3 (75.00)	5 (100.00)		
Changed	1 (25.00)	0 (0.00)	—	.44
Antipsychotics	16	16		
Unchanged	11 (68.75)	10 (62.50)		
Changed	5 (31.25)	6 (37.50)	0.76 (0.18 to 3.27)	>.99
Beta-blocking agents	15	15		
Unchanged	12 (80.00)	14 (93.33)		
Changed	3 (20.00)	1 (6.67)	3.50 (0.32 to 38.23)	.60

Note: Antidepressants included escitalopram, sertraline, paroxetine, fluoxetine, duloxetine, venlafaxine, desvenlafaxine, mirtazapine, vortioxetine, bupropion, agomelatine, amitriptyline, imipramine, milnacipran, tianeptine, and trazodone. Anxiolytics included alprazolam, etizolam, lorazepam, clonazepam, diazepam and buspirone. Hypnotics/sedatives included zolpidem, eszopiclone, zaleplon, melatonin and doxepin. Antipsychotics included aripiprazole, quetiapine, olanzapine, risperidone, and lurasidone. Beta-blocking agents included propranolol. Abbreviations: CI, confidence interval.
